# Experimental measurement of preferences in health care using best-worst scaling (BWS): theoretical and statistical issues

**DOI:** 10.1186/s13561-015-0077-z

**Published:** 2016-01-29

**Authors:** Axel C. Mühlbacher, Peter Zweifel, Anika Kaczynski, F. Reed Johnson

**Affiliations:** 1 Institute Health Economics and Health Care Management, Hochschule Neubrandenburg, Neubrandenburg, Germany; 2 Department of Economics, University of Zürich, Zürich, Switzerland; 3 Center for Clinical and Genetic Economics, Duke Clinical Research Institute, Duke University, Durham, USA

**Keywords:** Choice Experiments, Stated Preferences, Discrete Choice Experiments, Best-Worst Scaling, MaxDiff Scaling

## Abstract

For optimal solutions in health care, decision makers inevitably must evaluate trade-offs, which call for multi-attribute valuation methods. Researchers have proposed using best-worst scaling (BWS) methods which seek to extract information from respondents by asking them to identify the best and worst items in each choice set. While a companion paper describes the different types of BWS, application and their advantages and downsides, this contribution expounds their relationships with microeconomic theory, which also have implications for statistical inference. This article devotes to the microeconomic foundations of preference measurement, also addressing issues such as scale invariance and scale heterogeneity. Furthermore the paper discusses the basics of preference measurement using rating, ranking and stated choice data in the light of the findings of the preceding section. Moreover the paper gives an introduction to the use of stated choice data and juxtaposes BWS with the microeconomic foundations.

## Background

When searching for optimal solutions in health care, decision makers inevitably must evaluate trade-offs, which call for multi-attribute valuation methods [1]. Discrete-choice experiment (DCE) methods have proven to be particularly useful [2–6]. DCEs decompose choice alternatives into specific attributes or outcomes, permitting to identify the implicit decision weights survey respondents employ in making choices among combinations of health and healthcare outcomes [7, 8]. More recently, some researchers have proposed using best-worst scaling (BWS) methods which seek to extract additional information from respondents by asking them to identify the best and worst items in each choice set. While a companion paper (Mühlbacher et al. [1]) describes the different types of BWS and their advantages and downsides, this contribution expounds their relationships with microeconomic theory, which also have implications for statistical inference. It is structured as follows. Section 2 is devoted to the microeconomic foundations of preference measurement, also addressing issues such as scale invariance and scale heterogeneity. In Section 3, preference measurement using rating and ranking data is discussed in the light of the findings of the preceding section. Section 2 and Section 3 particularly are addressed to scholars in health sciences. After an introduction to the use of stated choice data in Section 4, BWS is juxtaposed with the microeconomic foundations previously laid out (in Section 5). This has consequences for experimental design which are spelled out in Section 6. Finally, Section 7 presents some conclusions and an outlook on future research.

### Microeconomic foundations

The objective of this section is to discuss concepts that are at the core of microeconomic theory but may be unfamiliar to readers with a health sciences background. These concepts will facilitate the assessment of BWS methods in Section 5.

#### Preferences and indifference curves

Individual preferences determine the relative perceived satisfaction obtainable from various attributes or outcomes of decisions. Preference relations are assumed to conform to basic requirements of logic and consistency. The term ‘utility’ denotes the mathematical representation of preference relations. Microeconomic theory assumes that decision makers select alternatives that maximize the value of their utility function, subject to resource constraints [9]. Such choices thus result in the highest obtainable level of subjective satisfaction.

**Fig. 1:**
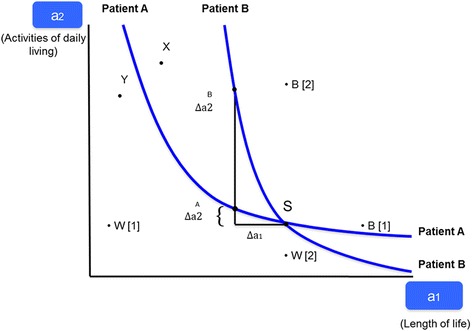
Preference elicitation with DCE

Preference relations can be described using indifference curves along which utility is held constant, implying that the decision maker is indifferent between different combinations of attributes. Indifference curves show how individuals evaluate subjective trade-offs among attributes. Figure 1 illustrates trade-offs between length of life and activities of daily living (an indicator of quality of life). The status quo (point *S*) represents the conventional standard of care, characterized by a long life (*a*_1_) but limitations on activities of daily living (*a*_2_). Two indifference curves pass through *S*, indicating combinations of attributes judged to yield the same level of utility for patients A and B. Patient B’s indifference curve has a steeper slope than that of patient A. This means that patient B places a relatively high value on length of life because he or she needs to obtain a comparatively high compensation in terms of activities of daily living $\varDelta {a}_2^B$ to accept a given reduction in length of life *Δa*_1_. In contrast, patient A places a relatively lower value on quality of life and thus has a flatter indifference curve indicating that a relatively small improvement $\varDelta {a}_2^A$ suffices to compensate him or her for the same reduction in length of life *Δa*_1_. Thus the slope of an indifference curve indicates the relative importance of an attribute and therefore the structure of an individual’s subjective preferences. The slope of the indifference curve, e.g. $\varDelta {a}_2^A$ / *Δa*_1_, is called the marginal rate of substitution.

#### Experiments to identify indifference curves

A controlled experiment with a sample of respondents can help identify the preferences of individuals such as patient A. Let survey respondents be of type A. Make them choose between the combination of attributes *X* (more activities of daily living *a*_2_, clearly shorter length of life *a*_1_) and the status quo *S*. If they choose *X*, then *X* evidently is better than *S* (see Fig. 1 again), implying that A’s indifference curve must pass below *X*. Next, the researcher mixes the two attributes again, resulting in the combination *Y*. Respondents now are asked to choose between the status quo *S* and *Y*. Assume that respondents prefer *S* this time. This means that their indifference curve passes above *Y*. By repeating this procedure (including using points B[1] and W[2] which will be of importance below), it is possible to identify the indifference curve. Its slope $\varDelta {a}_2^A/\varDelta {a}_1$ shows that for respondents like patient A, a given improvement in quality of life indicated by activities of daily living would have to be offset by a relatively large loss of life years. The preference relations of respondents like patient B are quite different. Notably, combination *X* lies below patient B’s indifference curve through the status quo point *S*, causing B to prefer the status quo. The reason is that B’s ratio $\Delta {a}_2^B/\varDelta {a}_1$is greater in absolute value than A’s, indicating that a given loss of life years must be compensated by a substantial improvement in quality of life $\Delta {a}_2^B$ for B’s utility to remain constant.

#### Deriving equivalents

Knowledge of the marginal rate of substitution between quality and quantity of life makes it possible to derive time equivalents for improvements in quality of life analogous to conventional time equivalents. In addition, DCE data can be used to identify the relative value of health outcomes in terms of other equivalents. For example, by replacing changes in length of life with changes in net income that are associated with a movement away from the status quo (see Fig. 1 again), one obtains the individual’s marginal willingness to pay for a small improvement in the quality of life. Longevity could also be replaced with the risk of a serious side effect to derive maximum acceptable risk for an increase in quality of life (e.g. brought about a therapy). Therefore, an individual’s risk-efficacy trade-off can be estimated; indeed, outcome equivalents can be derived for any continuous attribute.

Up to this point, focus has been on marginal changes in one attribute. However, the logic of the argument extends to discrete changes in treatment profiles that allow for combinations of changes in multiple attributes. Using DCE data to quantify preference relations among multiple attributes makes it possible to calculate time, money, or risk equivalents of changes in total utility between two treatment profiles or between a treatment profile and the status quo [10]. Therefore, a DCE can be used to evaluate both changes in individual attributes as well as combinations of attributes associated with decisions bearing on resource allocation.

#### Scale invariance

Basing the analysis on microeconomic theory has the crucial advantage of scale invariance. Metrics based on the slope of the indifference curve do not depend on the scaling of the attributes considered, rendering the interpretation of results invariant to scale. This can be shown as follows. Let *U* = *f* (*a*_1_, *a*_2_) be the utility function indicating an individual’s subjective valuation of attribute combinations. By definition, utility is constant along an indifference curve, so Δ*U* must be zero. As a change Δ*U* can only result from changes in the levels of attributes Δa_1_ and Δa_2_, one has

1 $$ \Delta \mathrm{U} = \frac{\partial f}{\partial {a}_1}\cdot {\Delta \mathrm{a}}_1+\frac{\partial \mathrm{f}}{\partial {\mathrm{a}}_2}\cdot {\Delta \mathrm{a}}_2=0 $$

with ∂*f*/∂*a*_1_ and ∂*f*/∂*a*_2_ indicating the marginal utility of unit changes in the respective attributes. By solving for Δa_2_/Δa_1_, one obtains the slope of the indifference curve (see Fig. 1 again),

2 $$ \frac{\Delta {a}_2}{\Delta {a}_1} = -\frac{\partial f/\partial {a}_2}{\partial f/\partial {a}_1} $$

Now let the utility function be transformed by any positive affine transformation *φ*(⋅). Such a transformation causes differences in attributes to have a stronger or weaker impact on utility, respectively. With *Ũ* = *φ*(*U*), the equation for the new indifference curve reads,

3 $$ \Delta \tilde{\mathrm{U}} = \frac{\partial \varphi }{\partial f}\cdot \frac{\partial f}{\partial {a}_1}\cdot {\Delta \mathrm{a}}_1+\frac{\partial \varphi }{\partial f}\cdot \frac{\partial \mathrm{f}}{\partial {\mathrm{a}}_2}\cdot {\Delta \mathrm{a}}_2=0 $$

This equation can again be solved for the slope *Δa*_2_/*Δa*_1_, resulting in

4 $$ \frac{\Delta {a}_2}{\Delta {a}_1} = -\frac{\partial \varphi /\partial f\cdot \partial f/\partial {a}_2}{\partial \varphi /\partial f\cdot \partial f/\partial {a}_1} = -\frac{\partial f/\partial {a}_2}{\partial f/\partial {a}_1} $$

Thus the slope of an indifference curve is not affected by a change in scaling. Note that this invariance result includes scalings that affect attributes differently as long as *φ*(⋅) is order-preserving, since these partial transformations can still be represented by a single *φ*(⋅) function.

#### Scale heterogeneity

The foregoing argument relates to scale transformations in the sense of order-preserving adjustments in the metric used to assign numbers to an individual utility function. As shown, such transformations have no effect on the shapes of individual indifference curves. However, empirical estimates are based on a sample of individuals, for whom a linear-additive specification of a random utility function is often postulated (see Section 5.1 below),

*U*_*ij*_ = *βa*_*ij*_ + $\frac{\varepsilon_{ij}}{\sigma }$ (5)

there *i* indexes individuals, *j* indexes choice alternatives, *a* is a vector of attributes that varies not only with choice alternatives *j* but also individuals *I* as soon as “net income” is an attribute (through the price attribute), β is a vector of marginal utilities corresponding to ∂*f*/∂*a*, *ε*_*ij*_ is an error term capturing the effect of unobserved factors, including idiosyncratic preferences for unobserved attributes, and σ is the scale of the error term determining its variance. This scale is not identified in most empirical models and usually normalized to 1 [11].

Suppose, however, that σ is individual-specific, so the scale actually is σ_i_. Replacing σ by σ_i_ in (5) and multiplying through by σ_i_, one obtains

6 $$ {\tilde{U}}_{ij}=\left(\beta {\sigma}_i\right){a}_{ij}+{\varepsilon}_{ij} $$

Thus the marginal utility parameters β are scaled up or down proportionally across individuals by σ_i_. Assuming σ_i_ = 1 for all individuals preserves the results (1–4) for a sample of individuals but neglects inherent heterogeneity across individuals. In principle, scale also could vary across choice sets *j* in a preference-elicitation survey because of learning and fatigue, sequence effects, and pairing of the same choice profile with other profiles so that

7 $$ {\tilde{U}}_{ij}=\left[\beta {\sigma}_{ij}(Z)\right]{a}_{ij}+{\varepsilon}_{ij} $$

where Z is a vector of covariates in the scale function. Thus estimated marginal utility parameters $\widehat{\beta}=\beta {\sigma}_{ij}(Z)$ potentially are confounded with scale effects both across individuals and within individuals across choice questions.

Some authors have argued that scale heterogeneity effectively invalidates all conventional approaches to estimating choice models [12]. Choice-modelling strategies such as the Generalized Multinomial Logit model offer a way to obtain separate estimates of taste and scale parameters [13]. Also, using a DCE to identify a single indifference curve (as predicated by microeconomic theory) eliminates scale differences both across attributes and individuals (see Section 5.3 below).

### Preference measurement using rating and ranking data

The rating procedure calls for indicating total utility on a categorical Likert or continuous visual-analogue rating scale. These scales usually have defined endpoints indicating minimum and maximum values [14]. While these preference measures are easy to obtain, they have methodological weaknesses. First, rating responses tend to discriminate poorly among categories, causing the values obtained to exhibit insufficient variability. Because there are no tradeoffs involved, respondents tend to ignore relative importance and say that everything is important, giving rise to ceiling effects [15]. In terms of Fig. 1, all the alternatives appear to lie on the same indifference curve, which results in illogical rankings of naturally ordered alternatives.

Second, responses often are influenced by social attitudes resulting in yea-saying and nay-saying bias. In the introductory example, type-A patients could be under the impression that length of life is valued very highly by society, causing them to overstate ratings for longevity. Arguably, yea-saying also could bias a DCE to the extent that respondents of type B opt too frequently for the alternative with a higher score for activities of daily living. However, DCEs usually call for choices between alternatives that differ in terms of several attributes, making it more difficult to identify socially acceptable alternatives.

Third, ratings are a difficult cognitive task, requiring evaluation of the intensity of preference on a predetermined numerical scale [14]. This can result in measurement error; it is much easier for respondents to say they prefer one alternative to another than to assign a number to the degree they prefer one alternative to another. In addition, cultural response styles may differ among countries [16]. Fourth, microeconomic theory postulates that respondents value attributes in relation to each other, as discussed in the context Fig. 1 [14, 17]. If the theory is descriptive of choice behaviour, the rating format will not produce reliable preference data [18].

More importantly, ratings assume utility to be a cardinal construct such that a given score measures the same utility across individuals and a given difference in score is associated with the same difference in utility under all circumstances. Yet for predicting choice, it is sufficient to compare alternatives in terms of “better” or “worse” (see Fig. 1 again). Therefore, utility is an ordinal construct in microeconomic theory. In return, the curvature of the indifference curve indicates that the ratio of marginal utilities is not a constant but depends on the individual’s position in attribute space [see eq. (4)], implying that the assumption of constant ß’s in eq. (5) can be a local approximation at best.

To illustrate the difficulty with ratings as a cardinal construct, assume that two alternatives have 50 and 60 points on the rating scale, respectively. In Fig. 1, let this 10 point difference correspond to the distance between points *X* and *Y*. For type-A patients, such a difference reflects a substantial difference in utility. Yet for type-B patients, the same numerical difference could indicate a small difference in utility since both points lie at a considerable distance from their indifference curve through the status quo *S*. For instance, let attribute *a*_1_ be associated with utility values 10, 20, and 30 and *a*_2_, with values 20, 40, and 60 indicating that the third alternative with values (30, 60) is preferred. Here, attribute *a*_2_ appears to be twice as important as *a*_1_. However, one could have assigned the values 10, 20, and 30 to attribute *a*_2_ as well, causing *a*_1_ and *a*_2_ to appear equally important. Yet the researcher would observe that the individual opts for the third alternative again, regardless of the numerical scaling of the attributes. This implies that it is impossible to infer an absolute scale from observed choices or to make inferences by comparing numerical values between individuals.

Therefore, microeconomic theory suggests that ranking, which respects the ordinal property of utility, is superior to rating in terms of validity and reliability of responses. Just stating that the alternative 30 is better than the alternative 20 which in turn is better than the 10 is sufficient [19]. Still, the challenge is to come up with a complete preference ordering over all outcomes of interest. While the ranking method is in agreement with microeconomic theory, it has several weaknesses. For researchers, designing a ranking task is far more demanding than a DCE; for respondents, it imposes a heavy cognitive burden. While it usually is relatively easy to rank alternatives at the top and bottom, ranking of alternatives in the middle of a long list has shown to be unreliable [20]. To avoid this, the number of alternatives must be small, which also limits the number of attributes (respondents would need to be presented with many alternatives for ensuring that each attribute is included at least once in a bundle).

### Preference measurement using stated choice data

Relying on stated choices is closest to everyday expression of preference; moreover, it is consistent with microeconomic theory. In the conventional DCE format respondents select only one alternative at a time, which provides no information about the ordering of alternatives not chosen. Because relatively little preference information is obtained from choices among two or three alternatives, it is necessary to have respondents answer a series of choice questions. A review of health DCEs found that the typical number of choice questions ranged from 8 to 12 [21]. Still, there are two situations where choice data can be uninformative about respondent preferences. They are uninformative when respondents either always pick the alternative with the better level of one attribute or always pick the status-quo or opt-out alternative. These choice patterns fail to provide the information about trade-offs that is required to estimate marginal rates of substitution. They imply that indifference curves are straight vertical or horizontal lines [18, 22]. Although indifference curves of this type are implausible, they may be the consequence of experimental design that does not offer sufficiently attractive alternatives to one featuring a strongly preferred attribute or to the status quo.

### Best-worst scaling

Several early contributions pointed out that some of the approaches described up to this point put high cognitive demands on respondents while others have weaknesses from the vantage point of measurement theory [23, 24]. As a response to these criticisms, BWS was developed in the late 1980s as an alternative to existing methods [25, 26]. Flynn (2010) distinguishes three cases of BWS which have in common that respondents, rather than just identifying the best alternative, simultaneously select the best and worst alternative from a set of three or more alternatives [27–29]. One of the three possible variants is very similar to DCEs, making it well anchored in microeconomic theory (for more descriptive detail, see the companion paper [1]). All variants have one thing in common: they require respondents to examine all alternatives comprising a choice scenario and to perform a dual choice by identifying not only the best but also the worst attribute, attribute level, or combination of multiple attribute levels [30, 31]. The resulting data thus identify pairs of alternatives with maximum differences in utility [32]. The remainder of this section is devoted to a critical discussion of the three variants of BWS in the light of microeconomic theory.

### Object case BWS

The first variant of BWS is the attribute or object case. It is the original form of BWS as proposed by Finn and Louviere [33], designed to determine the relative importance of attributes [29]. Accordingly, attributes have no (or only one) level, and choice scenarios differ merely in the particular subset of attributes shown. Respondents are asked to identify the most and least preferred attribute from the scenario list [28]. The number of scenarios required to identify a complete ranking depends on the number of attributes. The BWS object case originally was conceived as a replacement of traditional methods of preference measurement such as ratings and Likert scales [29].

The object case avoids problems that occur with rating scales because it normalizes all relative-importance weights to the (0,1) interval and thus eliminates scale artefacts as shown in equations (5–7). In principle, therefore, it facilitates valid comparisons of preference [34]. It also reduces social-desirability bias because it makes respondents evaluate trade-offs between attributes [35]. For this reason, ties in orderings are rare compared to rating data. These advantages have motivated the use of the object case for assessing health states, quality of life, and worker satisfaction [36, 37].

However, the object scaling variant of BWS lacks accuracy and discriminating power. The example of two different patient types (“patient A” and “patient B”) of Fig. 1 illustrates this problem. Both types need to choose between alternative treatments. As before, let the two attributes be length of life (*a*_1_) and improvement in activities of daily living (*a*_2_). However, object case BWS forces them to take on just two values, 0 (not present in scenario) and 1 (present in scenario).

**Fig. 2:**
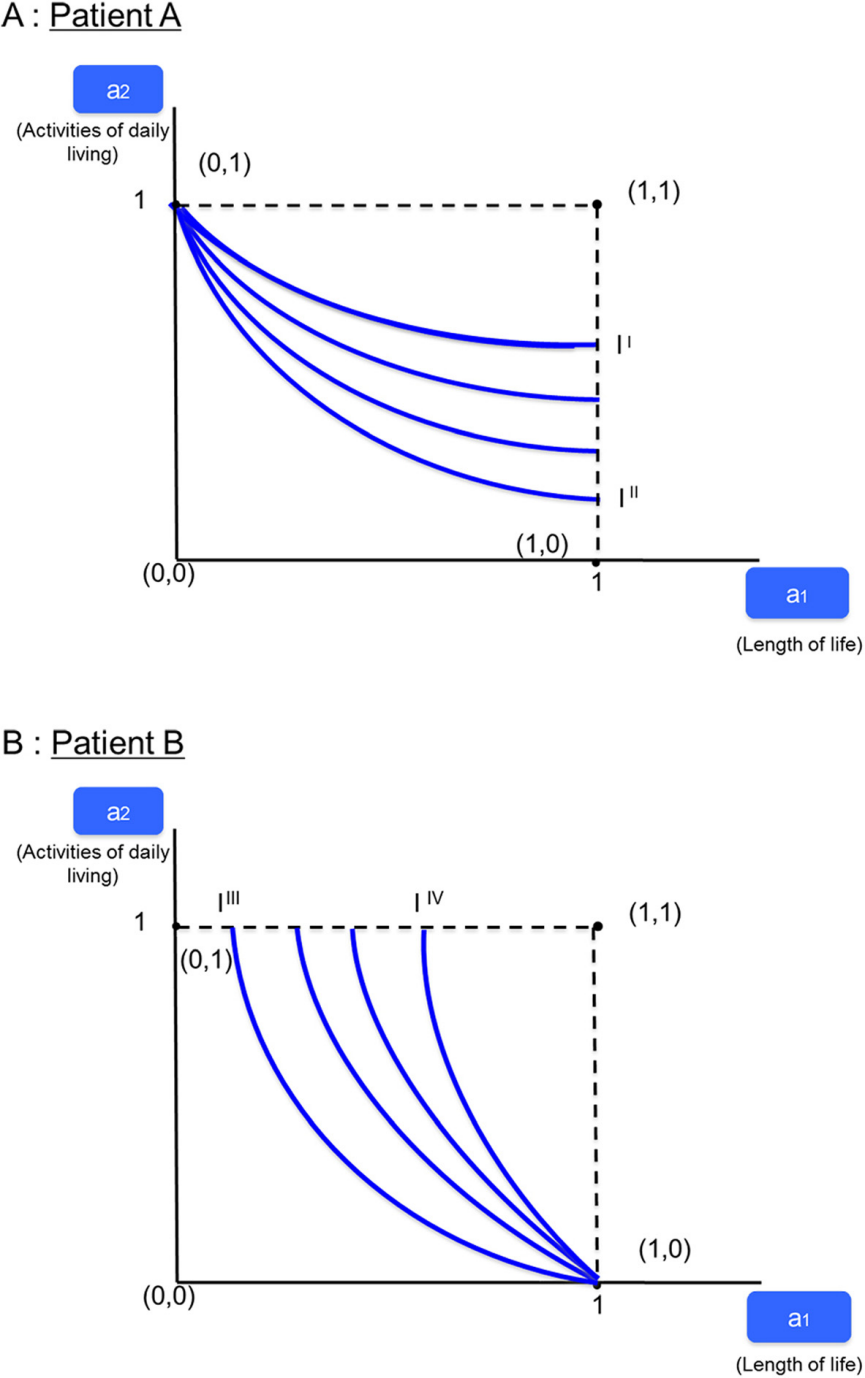
Preference Elicitation with BWS Object Case

As shown in Fig. 2, this reduces the set of possible alternatives to four, represented by the points (0,0), (0,1), (1,0), and (1,1). As in Fig. 1, the objective is to determine the slope of the indifference curve, reflecting the relative importance of the two attributes. However, point (0,0) mirroring “worst” is not informative since no indifference curve can possibly go through it (any other bundle of attributes is better). The same is true of point (1,1) since all other bundles are worse.

Panel A of Fig. 2 depicts patient A, whose indifference curve has relatively flat slope, indicating that activities of daily living are relatively important. Only point (0,1) qualifies as the origin of the indifference curve because point (1,0), associated with a total loss of activities of daily living, is unacceptable for this type of respondent. This leaves all indifference curves bounded by I’ and I”in the possible set. The “best” choice can only be (1,1), and the “worst” one, (0,0). With these weak restrictions, object case BWS fails to limit the set of admissible indifference curves and their slopes.

Panel B of Fig. 2 depicts patient B, whose indifference curve has a relatively steep slope, indicating that longevity is important compared to activities of daily living. As in panel A, points (0,0) and (1,1) cannot be the origin of an indifference curve. Point (0,1) is unacceptable for someone with a strong preference for gaining life years. This leaves point (0,1), from which any indifference curve can originate as long as its slope (in absolute value) is less than that of patient A depicted in panel A. The set of admissible indifference curves therefore is bounded only by I”’ and I’^*v*^; it cannot be identified any further using object case BWS.

Object case BWS thus has a serious drawback since its attributes take on only the value 1 (present) or 0 (not present) rather than a set of levels. Therefore, researchers cannot know what level respondents impute to a specific attribute relative to other attributes. Furthermore, the slopes of the indifference curves pertaining to respondents with different preferences cannot be identified with any precision. Object case data thus do not permit to determine the relative importance of attributes or to compare it between respondents with differing preferences.

### Profile case BWS

The second BWS variant is the profile case [38]. Here, the same attributes appear in each scenario but differ in terms of their levels, with respondents identifying both the “best” and “worst” attribute level in each scenario shown [27]. Profile case BWS has advantages relative to both the object case and DCEs. In contrast to object case BWS, respondents explicitly value attribute levels, making choices much more transparent and informative. Compared to a DCE, respondents evaluate only one profile scenario at a time, which obviates correctly combining profiles. Also, the cognitive burden of the preference-elicitation task may be reduced, permitting the number of attributes to be increased [12].

However, the profile case has three weaknesses. First, at least in its so-called maxdiff formulation, it assumes a cardinal utility function, which causes the difficulties expounded in Section 3 above. In particular, the maxdiff model assumes that *BW*_*X*_ (*x*, *y*), *x* ≠ *y*, is proportional to *b*(*x*)/*b*(*y*), with *x* and *y* denoting two alternatives (points in Fig. 1), *BW*_*X*_ denoting the best-worst distance, and *b(x)* and *b(u)* symbolizing two utility values that are defined by *u*(*x*) = log *b*(*x*) and *u*(*y*) = log *b*(*y*), respectively. Evidently, the best-worst distance is expressed in terms of cardinal utility [12, 32].

**Fig. 3:**
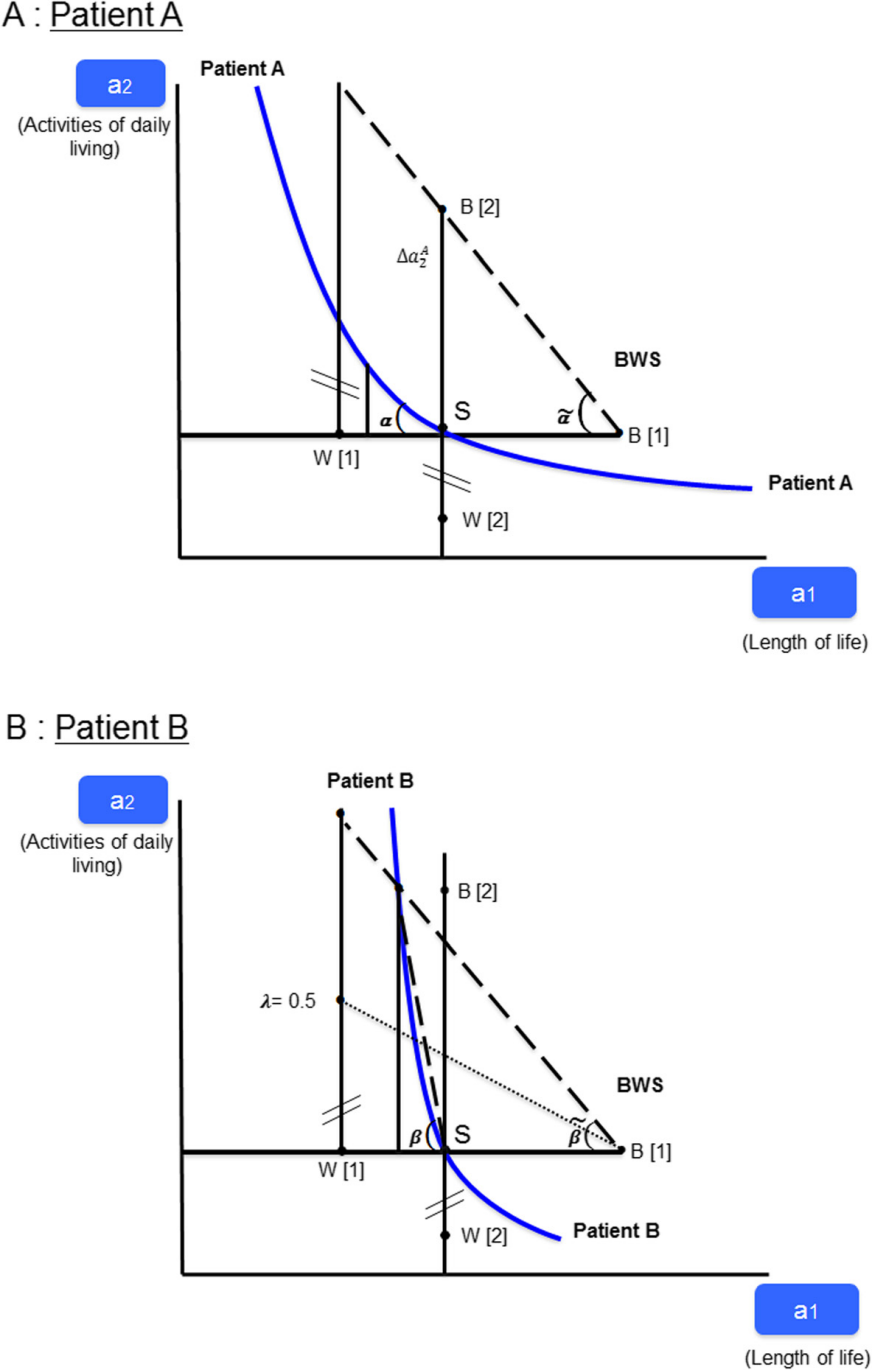
Preference Elicitation with the BWS Profile Case

Second, the maxdiff approach fails to determine the relative importance of attributes. Recall from Fig. 1 that the slope of the indifference curve indicates the relative importance for small changes in levels. For example, in Fig. 1 let B[1] be the “best” level of attribute *a*_1_, and W[1], the “worst”, as identified by patient A. This valuation holds all other attributes constant at a given level, which must be specified in a well-designed experiment. For simplicity, the status-quo value is used here. Accordingly, W[1] and B[1] lie on a horizontal line through point *S* (the status quo), while W[2] and B[2] lie on a vertical line through *S.* The relative importance of an attribute is indicated by the angle α, which shows the sacrifice of length of life that would be acceptable in return for improved activities of daily living (see panel A of Fig. 3).

According to profile case BWS, this angle is given by the ratio of maxdiff values, i.e. {B[2]–W[2]} / {B[1]–W[1]} (note that in panel A of Fig. 3, the vertical distance {B[2]–W[2]} has to be moved to originate from W[1] in order to determine the angle). This ratio defines the (tangent of) angle *ã*. However, *ã* differs substantially from the true angle a, causing the researcher to erroneously conclude that length of life is very important to a person like patient A because any loss in this attribute would have to be highly compensated by an improvement in activities of daily living.

Third, the profile case also discriminates poorly among respondents with different preferences. Note that while the indifference curves of patients A and B have different slopes, the two respondents could agree on their best and worst values both with regard to attribute *α*_1_ (given *α*_2_ is at its status-quo-level) and *α*_2_ (given *α*_1_ is at its status-quo level). Thus, in panel B of Fig. 3 profile case BWS yields the angle $\tilde{\beta}$, which clearly exceeds the true slope *β* of the indifference curve in the neighbourhood of point *S*. However, differences in utility are likely to differ between individuals. For instance, let the distance between B[2] and W[2] be associated with one utility difference for patient B but another utility difference for patient A. This is equivalent to a transformation *φ*(⋅) of utility from *U* to *Ũ*, as discussed in Section 2 above. In particular, let the difference in utility for patient B be only one-half of that of patient A (λ = 0.5). This causes the measured angle $\tilde{\beta}$ to become 0.5 $\tilde{\beta}$ -- much smaller than *β*. Comparing 0.5 $\tilde{\beta}$ (punctuated line) with the angle *ã* > 0.5 $\tilde{\beta}$ of panel A of Fig. 3 (dashed line), the researcher would erroneously conclude that length of life is more important to patient A than to patient B because A seemingly needs to be more highly compensated by improvement in activities of daily living. Thus profile case BWS may result in wrong inferences about differences in preferences if unobserved heterogeneity is present. This effectively is the critique BWS proponents level against DCE estimates. However, as long as the objective is to estimate marginal rates of substitution (which it should be), the two pertinent *U*_*ij*_ = (*βσ*_*i*_)*a*_*ij*_ + *ε*_*ij*_ value in eq. (6) are divided by each other, leaving the ratio of *β* ' *s* unaffected. Only if *σ*_*i*_ is a function of individual covariates *Z* [as in eq. (7)] does heterogeneity cause bias in the estimation of marginal rates of substitution based on a DCE.

### Multiprofile case BWS

The third BWS variant is the multiprofile case [29, 39]. Contrary to the two previous cases, respondents repeatedly choose between alternatives defined by full outcome profiles that include all the attributes set at different levels in a sequence of choice sets. Thus, the multiprofile case BWS amounts to a best-worst discrete-choice experiment (BWDCE). A BWDCE extracts more information from a choice scenario than a conventional DCE because it asks not only for the “best” (i.e. most preferred) but also the “worst” (least preferred) alternative.

Multiprofile case BWS has been used but rarely for preference measurement in health care, although BWDCE results are as reliable as those from conventional DCEs [40]. This is to be expected in view of Fig. 1; indeed, since the objective of a DCE is to identify an indifference curve, it is questionable how ‘best’ and’worst’ alternatives do add more information. One might argue, that ‘best’ and’worst’ are simply alternatives lying above and below the indifference curve (through the status quo point). Therefore, the claim that BWDCE yields more accurate measurements thanks to the additional information extracted lacks a theoretical foundation [12]. BWDCE just adds more information with regard to a single choice set.

Another important distinction is that BWDCEs call for a judgment as to which alternatives are “best” and “worst”. In contrast, DCEs ask which alternative the respondent would actually choose among those available. Conceivably, respondents might judge an alternative “best” they would not end up choosing. This could easily occur if the design lacks a price attribute, whereas respondents become aware of it when “choice” is mentioned in the DCE. Therefore, BWSDCE judgment data need not have the same utility-theoretic properties as DCE data. Nevertheless, BWSDCE data often are analyzed as if they were DCE data with additional information about preferences.

### Experimental measurement

#### Attributes and levels

Several methods are available for choosing attributes that can be used in combination [41]. Direct approaches include the elicitation technique, the repertory grid method as well as directly asking for attributes relative subjective importance [42]. All essential attributes should appear in the choice scenarios to avoid specification error in estimating the utility function [5, 26].

With the relevant attributes identified, their levels need to be defined (at least for profile and multiprofile BWS). Their ranges represent the perceived differences in respondent utility associated with the most and least preferred level. However, the reverse is not true: A respondent’s maximum difference in utility may fall short of or exceed the spread between levels as imposed by the experiment. Also, requiring attribute levels to be realistic appears intuitive. Yet, the experiment also calls for a spreading of levels, especially in the price attribute (assuming willingness-to-pay values are to be calculated). The reason becomes evident from considering Fig. 1 again. There, the unknown indifference curve can be interpolated best if respondents "jump back and forth" across it. Thus, the researcher must trade off two objectives. On the one hand, a data set as complete as possible is desirable, calling for attribute levels to be in a realistic range. On the other hand, it is important to be able to estimate the regression parameter associated with the price attribute as precisely as possible because being an estimate of the (negative of) marginal utility of income, income, − ∂*f*/∂*a*_1_, it enters the calculation of all willingness-to-pay values.

In principle, attributes can be measured on both nominal and ordinal scales. However, qualitative (nominally or ordinally scaled) descriptions provide respondents with room for interpretation. This can bias the results because of ambiguity, as was seen in the discussion of profile case BWS, by simply modifying the vertical distance {B[2]–W[2]} in Fig. 3. This would reflect failure of the respondent to correctly locate e.g. point B[2] in attribute space. Respondents also may reinterpret numerical levels by recoding them qualitatively as low, medium, and high in an attempt to simplify comparisons.

#### Experimental design

Survey design involves the construction of scenarios comprising combinations of attributes or attribute levels. As in the case of a DCE, there are several options available for BWS. From a complete list of possible combinations, suitable designs can be created manually by judiciously balancing several criteria, viz. the number of scenarios involving high and low (assumed) utility values, low correlation of attributes (orthogonality), balanced representation, and minimum overlap of levels [43]. If the reduced number of choice scenarios to be presented to respondents turns out to be still excessive, design blocks have to be created.

A frequently used alternative is the Balanced Incomplete Block Design (BIBD) [44]. For guidance concerning creation, analysis and operationalization of manual designs, the main reference is Cochran and Cox (1992), who created a multitude of ready-to-use BIBDs [45]. Ways to increase design efficiency are described in Chrzan and Orme (2000) and Louviere et al. (2000) [46, 47]. More recently, optimal and near-optimal designs complementing the manual approach have been developed [48].

Rather than manually developing a design, researchers can use automated (often computerized) procedures. For example, the software package SAS offers several search algorithms to determine the most efficient design of a given experiment [43]. However, efficient designs might result in biased estimates owing to some respondents using simplistic heuristics [49]. As shown in Flynn et al. (2015) higher efficiency in the design can be associated with smaller regression coefficients, suggesting either weaker preferences or lower choice consistency [49].

Simple orthogonal main-effect design plans (OMEPs) are available as well (e.g. in SPSS). Easy to use, they have been popular in BWS. However, OMEPs have the disadvantage that they do not allow marginal rates of substitution to depend on the level of attributes. This contradicts the convexity of the indifference curve. For example a loss in terms of activities of daily living needs to be offset only slightly by more of another attribute as long as the status quo contains much of it but needs to be highly compensated when it is scarce (indicated by points to the left of *S* in Fig. 3). In a (linearized) utility function *U* = *f*(*a*_1_, *a*_2_), this calls for interaction terms of the type (*a*_1_ ⋅ *a*_2_),

8 $$ U={\gamma}_0+{\gamma}_1\cdot {a}_1+{\gamma}_2\cdot {a}_2+{\gamma}_3\cdot \left({a}_1\cdot {a}_2\right)+\epsilon $$

such that, evaluated at the expected value of the disturbance term, *E*(*ϵ*) = 0,

9 $$ \frac{\partial U}{\partial {a}_1}={\gamma}_1+{\gamma}_3\cdot {a}_2 $$

and

10 $$ \frac{\partial U}{\partial {a}_2}={\gamma}_2+{\gamma}_3\cdot {a}_1 $$

*Z*_*i*_ This means that the marginal utility of an attribute depends on values of other attributes, a property that cannot be represented by an orthogonal design. Note also that by including interaction terms of the type (*a*_*j*_ ⋅ *Z*_*i*_), marginal utility and hence the marginal rate of substitution can be made to depend on an individual characteristic (for an application to individuals with and without chronic conditions, see e.g. Mc Neil Vroomen & Zweifel 2011 [4]). This serves to reduce the scope of preference heterogeneity to truly unobserved influences.

#### Statistical inference

In reality, researchers cannot know all determinants of utility; they must accept that observed choices contain a random element. In addition, respondents make errors in making hypothetical choices, as they do in their daily lives.

Random utility theory, developed by McFadden (1974, 1986), permits modelling decisions as a stochastic process [50, 51]. This model assumes maximization of expected utility (i.e. on average, after many repetitions) [52]. This is a much weaker behavioral standard than traditional utility maximization because it allows for respondents to be off target in any single choice, but on target on average.

Suppressing the index denoting the individual, equation (11) denotes the empirical indirect utility function for continuous or categorical attributes, where *U*_*j*_ is the individual’s utility for alternative *j*, *V*_*j*_ is the deterministic component, and *ε*_*j*_ is the random component,

11 $$ {U}_j = {V}_j + {\varepsilon}_j,\;j=1, \dots,\ J $$

The error term is assumed to follow a Gumbel or Type 1 extreme-value distribution, with expected value zero and constant variance. However, the usual independence assumption does not hold in this case because the same individual makes a series of evaluations during the experiment. For instance, someone who tends not to discriminate between bad alternatives is likely to commit the same type of error in the maxdiff procedure (the maxdiff procedure calls for identifying the maximum difference in utility, see Section 5.2 above). Thus, as shown in equations (5) to (7), the error term should include a scale function with arguments *Z* to account for scale variation among respondents, among attributes, across questions, and to accommodate sequence effects,

12 $$ {U}_j = {V}_j+\frac{\varepsilon_j}{\sigma (Z)},j=1, \dots,\ J $$

To simplify the discussion, assume σ(*Z*) = 1. It follows that the random utilities of the best and worst alternatives are

13 $$ {U}_b={V}_b+{\epsilon}_b $$

14 $$ {U}_w=-{V}_w-{\epsilon}_w, $$

respectively. According to the random utility model, the alternatives with the highest and lowest utility have the highest probability of being chosen as the best and worst alternatives,

15 $$ P(b)=P\left({U}_b=\underset{j\in C}{ \max}\left\{{U}_j\right\}\right), $$

$$ P(w)=P\Big({U}_w={ \max}_{j\in C}\left\{-{U}_j\right\} $$

and

16 $$ P\left(b,w\Big|C\right)=P\left({U}_b>{U}_j>{U}_w\right),\forall\ j\in {C}_{-\left\{b,w\right\}},\ b\ne w $$

where *C*_− {*b*,*w*}_ is the choice set *C* without alternatives *b* and *w*.

For the MNL choice model, the probability *P*_*B*_ of choosing alternative *j* as “best” from choice set *C* is given by

17 $$ P\left(b\Big|C\right)=\frac{e^{V_b}}{{\displaystyle {\sum}_{j\in C}}\ {e}^{V_j}} $$

However, BWS requires a dual choice of both the best and the worst alternative. Conventional MNL cannot be applied to BWS without modification because it deals only with the choice of one alternative [31]. One solution is to split the two choices into two independent decisions, as in rank or exploded logit analysis where the probability of choosing alternative *w* as “worst” is based on the alternatives remaining after alternative *b* is removed. Then, the joint probability of the two choices *P(b,w|C)* is the product of the individual probabilities,

18 $$ P\left(b,w\Big|C\right)=P\left(b\Big|C\right)\cdot P\left(w\Big|{C}_{-b}\right) = \frac{e^{V_b}}{{\displaystyle {\sum}_{j\in C}}\ {e}^{V_j}}\cdot \frac{e^{-{V}_w}}{{\displaystyle {\sum}_{k\in {C}_{-b}}}\ {e}^{-{V}_k}} $$

Alternatively, the maxdiff model assumes that respondents choose the best-worst pair out of all possible ordered pairs from the scenario with the greatest utility difference,

19 $$ \varDelta {U}_{bw}={V}_b-{V}_w+{\mathrm{\mathcal{E}}}_b-{\mathrm{\mathcal{E}}}_w, $$

resulting in the joint probability

20 $$ P\left(b,w\Big|C\right) = \frac{e^{\left({V}_b-{V}_w\right)}}{{\displaystyle {\sum}_{j\in C}}{\displaystyle {\sum}_{k\in C}}\ {e}^{\left({V}_j-{V}_k\right)}} $$

## Conclusions and outlook

While object case BWS and profile case BWS have been found to have weakness, multiprofile case BWS is in accordance with microeconomic theory. The demonstrated problems associated with BWS are particularly severe when individual’s preferences are not homothetic (introducing within-individual heterogeneity) and if preferences between individuals are heterogeneous. Moreover, it has been shown to provide results of comparable reliability as DCEs, regardless of design and sample size [28, 39]. Thus multiprofile case BWS (also known as DCEBWS), is best viewed as a refinement of the conventional DCE which opens up new opportunities in health economics and health services research. In particular, extracting additional information about preference from each respondent facilitates assessment of preference heterogeneity among respondents through the use of interaction terms involving individual characteristics in the random utility function to be estimated.

Physicians, researchers, and regulators often are poorly informed about the advantages and limitations of stated-preference methods. Despite the increased commitment to patient-centered healthcare, healthcare decision makers do not fully realize that knowledge of the subjective relative importance of outcomes to those affected is needed to maximize the health benefits of available healthcare technology and resources. Therefore, the collection of preference data that can measure preferences and differences in preferences in a valid way using DCEs and DCEBWS is of decisive importance for health economics and health services research.
